# Supplementary material for: A new moradisaurine captorhinid reptile (Amniota: Eureptilia) from the upper Permian of India
Source: PeerJ. 2024 Nov 14;12:e18394. doi: 10.7717/peerj.18394 (PMC11569782; doi:10.7717/peerj.18394)
Supplement: Supplemental Information 2 [file peerj-12-18394-s002.pdf]

Supplemental 2

```
P A U P *
Version 4.0a (build 169) for Macintosh (X86) (built on Feb 10 2021 at 16:12:22)
Monday, May 13, 2024 at 10:33:51 AM Atlantic Daylight Time

Running on Intel(R) Core(TM) i7-5650U CPU @ 2.20GHz
    Current processor contains 2 CPU cores on 1 socket (hyperthreaded to 4 logical cores)
    Executable built for IA-32 architecture (64-bit word length)
    Compiled using Intel compiler (icc) 11.1.0 (build 20091012)
    SSE vectorization enabled
    SSSE3 instructions supported
    Multithreading enabled using Pthreads

Processing of file "IndosauriscusMatrix.nex" begins...

Data matrix has 24 taxa, 79 characters
Valid character-state symbols: 01234
Missing data identified by '?'
Gaps identified by '-'
Case significant for alphabetic character-state symbols

paup> hsearch;

Heuristic search settings:
  Optimality criterion = parsimony
    Character-status summary:
      Of 79 total characters:
        7 characters are of type 'ord' (Wagner)
        72 characters are of type 'unord'
        All characters have equal weight
        All characters are parsimony-informative
    Gaps are treated as "missing"
    Multistate taxa interpreted as uncertainty
  Starting tree(s) obtained via stepwise addition
    Addition sequence: simple (reference taxon = Protorothyris)
    Number of trees held at each step = 1
  Branch-swapping algorithm: tree-bisection-reconnection (TBR) with reconnection limit =
8
    Steepest descent option not in effect
  Initial 'Maxtrees' setting = 100
  Branches collapsed (creating polytomies) if maximum branch length is zero
  'MulTrees' option in effect
  No topological constraints in effect
  Trees are unrooted

Heuristic search completed
  Total number of rearrangements tried = 16539
  Score of best tree(s) found = 236
  Number of trees retained = 4
  Time used = 0.01 sec (CPU time = 0.01 sec)

paup> contree;

Tree length = 236
Consistency index (CI) = 0.4449
Homoplasy index (HI) = 0.5551
Retention index (RI) = 0.6888
Rescaled consistency index (RC) = 0.3065
```

Supplemental 2

Strict consensus of 4 trees:

```
/---------------------------------------------------------------------------------------------------------------------------------- Protorothyris
|
|       /-------------------------------------------------------------------------------------------------------------------------- Paleothyris
|       |
|       |       /------------------------------------------------------------------------------------------------------------------ Thuringothyris
|       |       |
|       |       |       /---------------------------------------------------------------------------------------------------------- Euconcordia
|       |       |       |
\-------+       |       |                                                                                                 /-------- Romeria prima
        |       |       |                                                                                         /-------+
        |       |       |                                                                                         |       \-------- Romeria texana
        |       |       |                                                                                 /-------+
        |       |       |                                /------------------------------------------------+       \---------------- Reiszorhinus
        |       |       |                                |                                                |
        \-------+       |                                |                                                \------------------------ Protocaptorhinus
                |       |                                |
                |       |                                |                                                                /-------- Ca. laticeps
                |       |                                |                                                                |
                |       |                        /-------+       /--------------------------------------------------------+-------- Ca. aguti
                |       |                        |       |       |                                                        |
                |       |                        |       |       |                                                        \-------- Ca. magnus
                |       |                        |       |       |
                |       |                        |       |       |       /--------------------------------------------------------- Labidosaurus
                |       |                        |       |       |       |
                \-------+                        |       \-------+       |                                                /-------- Labidosaurikos
                        |                        |               |       |                                        /-------+
                        |                        |               |       |                                        |       \-------- Rothianiscus
                        |                        |               |       |                                        |
                        |                        |               |       |                                        |       /-------- Captorhinikos
                        |                        |               |       |                                /-------+-------+
                        |                /-------+               \-------+                                |       |       \-------- Sumidadectes
                        |                |       |                       |                        /-------+       |
                        |                |       |                       |                        |       |       \---------------- Gansurhinus
                        |                |       |                       |                        |       |
                        |                |       |                       |               /--------+       \------------------------ Moradisaurus
                        |                |       |                       |               |        |
                        |                |       |                       |       /-------+        \-------------------------------- Indosauriscus
                        |        /-------+       |                       |       |       |
                        |        |       |       |                       \-------+       \----------------------------------------- Tramuntanasaurus
                        |        |       |       |                               |
                        |        |       |       |                               \------------------------------------------------- Rhodotheratus
                        |        |       |       |
                        \--------+       |       \--------------------------------------------------------------------------------- Saurorictus
                                 |       |
                                 |       \----------------------------------------------------------------------------------------- Rhiodenticulatus
                                 |
                                 \------------------------------------------------------------------------------------------------ Opisthodontosaurus
```

```
paup> bootstrap nreps=1000
paup> set;

Maxtrees will be automatically increased by 100 when the limit is hit
;

Bootstrap method with heuristic search:
  Number of bootstrap replicates = 1000
  Starting seed = generated automatically
  Number of characters resampled in each replicate = 79
  Optimality criterion = parsimony
    Character-status summary:
      Of 79 total characters:
        7 characters are of type 'ord' (Wagner)
        72 characters are of type 'unord'
        All characters have equal weight
        All characters are parsimony-informative
    Gaps are treated as "missing"
    Multistate taxa interpreted as uncertainty
  Starting tree(s) obtained via stepwise addition
    Addition sequence: simple (reference taxon = Protorothyris)
    Number of trees held at each step = 1
  Branch-swapping algorithm: tree-bisection-reconnection (TBR) with reconnection limit =
8
    Steepest descent option not in effect
  Initial 'Maxtrees' setting = 100000
  Branches collapsed (creating polytomies) if maximum branch length is zero
  'MulTrees' option in effect
  No topological constraints in effect
  Trees are unrooted

  1000 bootstrap replicates completed
  Time used = 00:01:52 (CPU time = 00:01:54.2)
```

Supplemental 2

Bootstrap 50% majority-rule consensus tree

```
/----------------------------------------------------------------------------------------------------------------------------- Protorothyris(1)
|
|                    /-------------------------------------------------------------------------------------------------------- Paleothyris(2)
|                    |
|                    |                    /----------------------------------------------------------------------------------- Thuringothyris(3)
|                    |                    |
\--------------------+                    |                    /-------------------------------------------------------------- Euconcordia(4)
                     |                    |                    |
                     |                    |                    |                                         /-------------------- Romeria prima(5)
                     |                    |                    |                    /---------67---------+
                     \---------97---------+                    |                    |                    \-------------------- Romeria texana(6)
                                          |                    |                    |
                                          |                    |                    +----------------------------------------- Protocaptorhinus(7)
                                          |                    |                    |
                                          |                    |                    +----------------------------------------- Rhiodenticulatus(8)
                                          |                    |                    |
                                          |                    |                    +----------------------------------------- Saurorictus(9)
                                          |                    |                    |
                                          \---------90---------+                    |
                                                               |                    |                    /-------------------- Ca. laticeps(10)
                                                               |                    |                    |
                                                               |                    +---------76---------+-------------------- Ca. aguti(11)
                                                               |                    |                    |
                                                               |                    |                    \-------------------- Ca. magnus(12)
                                                               |                    |
                                                               |                    +----------------------------------------- Labidosaurus(13)
                                                               |                    |
                                                               |                    |                    /-------------------- Labidosaurikos(14)
                                                               |                    |                    |
                                                               \---------68---------+                    +-------------------- Moradisaurus(15)
                                                                                    |                    |
                                                                                    |                    +-------------------- Rothianiscus(16)
                                                                                    |                    |
                                                                                    |                    +-------------------- Captorhinikos(17)
                                                                                    +---------59---------+
                                                                                    |                    +-------------------- Gansurhinus(18)
                                                                                    |                    |
                                                                                    |                    +-------------------- Sumidadectes(19)
                                                                                    |                    |
                                                                                    |                    +-------------------- Tramuntanasaurus(23)
                                                                                    |                    |
                                                                                    |                    \-------------------- Indosauriscus(24)
                                                                                    |
                                                                                    +----------------------------------------- Reiszorhinus(20)
                                                                                    |
                                                                                    +---------------------------------------- Opisthodontosaurus(21)
                                                                                    |
                                                                                    \----------------------------------------- Rhodotheratus(22)
```

Bipartitions found in one or more trees and frequency of occurrence (bootstrap support values):

```
         1         2   2
123456789012345678901234          Freq        %
-----------------------------------------------
..**********************        967.32   96.73%
...*********************        901.98   90.20%
.........***............        760.61   76.06%
....********************        677.25   67.73%
....**..................        666.99   66.70%
.............******...**        591.88   59.19%
............*******...**        484.86   48.49%
................*.*.....        407.82   40.78%
.............*.*........        373.49   37.35%
.........**.............        354.18   35.42%
.........**********.****        342.34   34.23%
.............******..***        315.37   31.54%
.........**********...**        307.18   30.72%
.............******.....        295.47   29.55%
............*******..***        280.35   28.04%
....**.............*....        256.33   25.63%
.............***.*....**        251.53   25.15%
..............**........        216.51   21.65%
.........**********..***        215.15   21.51%
.............******....*        195.28   19.53%
....****************.***        189.08   18.91%
....***.................        188.54   18.85%
....................**..        185.82   18.58%
.....................**.        184.95   18.50%
.............******...*.        173.73   17.37%
.............***.*......        167.50   16.75%
...............*.......*        167.35   16.73%
....***............*....        167.30   16.73%
.............***.*.....*        166.87   16.69%
.......**...............        160.28   16.03%
........***********.****        150.82   15.08%
....***.****************        148.90   14.89%
............*******.****        144.21   14.42%
..............**.......*        143.10   14.31%
................***.....        132.56   13.26%
```

```
......................**        132.00   13.20%
.............******.****        130.00   13.00%
..............*..*......        122.17   12.22%
....****************..**        121.51   12.15%
......******************        119.34   11.93%
.........**********.*.**        116.52   11.65%
.................**.....        111.76   11.18%
............*******....*        108.55   10.85%
....*****************.**        107.57   10.76%
....*.*.................        105.89   10.59%
.....................***        104.34   10.43%
.............*.****.....        103.65   10.36%
......*************.****        102.61   10.26%
.............***.**...**        100.15   10.01%
.........*.*............         97.40    9.74%
......**************.***         94.39    9.44%
.......************.****         93.80    9.38%
....***.************..**         92.46    9.25%
.............***.*...***         90.32    9.03%
..............**.*......         89.53    8.95%
.................*....*.         85.54    8.55%
......*************..***         84.29    8.43%
....***..***************         80.85    8.09%
.............*****....**         78.57    7.86%
.............*.**.......         76.35    7.63%
..............**.*....**         75.97    7.60%
....***..***********..**         75.20    7.52%
........***********..***         74.47    7.45%
......*.*...............         73.44    7.34%
.............***.......*         72.95    7.29%
.......************..***         67.54    6.75%
....***.************.***         63.66    6.37%
...*****.***************         63.07    6.31%
............*******.*.**         62.01    6.20%
.........***........*...         60.32    6.03%
..........**............         60.21    6.02%
......*************...**         59.63    5.96%
.............*..***.....         59.37    5.94%
..............**.*...***         58.93    5.89%
.............*.*.*......         57.58    5.76%
....****.***************         57.09    5.71%
.............**.***.....         55.98    5.60%
.......************...**         54.71    5.47%
.............***......**         54.20    5.42%
............****.**....*         54.05    5.40%
...*................*...         52.66    5.27%
......*..**********.****         52.24    5.22%
.............***.**.....         52.21    5.22%
.............***.**....*         51.99    5.20%
......*.***********.****         51.84    5.18%
.............*****...***         51.08    5.11%

1333 groups at (relative) frequency less than 5% not shown


Character types changed:
  Of 79 total characters:
    7 characters are of type 'ord' (Wagner)
    72 characters are of type 'unord'
    All characters have equal weight

paup> ctype Ord:6-7 9-10 12 45 67;

paup> hsearch;

Heuristic search settings:
  Optimality criterion = parsimony
    Character-status summary:
      Of 79 total characters:
        7 characters are of type 'ord' (Wagner)
        72 characters are of type 'unord'
        All characters have equal weight
```

```
        All characters are parsimony-informative
    Gaps are treated as "missing"
    Multistate taxa interpreted as uncertainty
  Starting tree(s) obtained via stepwise addition
    Addition sequence: simple (reference taxon = Protorothyris)
    Number of trees held at each step = 1
  Branch-swapping algorithm: tree-bisection-reconnection (TBR) with reconnection limit =
8
    Steepest descent option not in effect
  Initial 'Maxtrees' setting = 100
  Branches collapsed (creating polytomies) if maximum branch length is zero
  'MulTrees' option in effect
  No topological constraints in effect
  Trees are unrooted

Heuristic search completed
  Total number of rearrangements tried = 16539
  Score of best tree(s) found = 236
  Number of trees retained = 4
  Time used = 0.01 sec (CPU time = 0.01 sec)

paup> contree;

Strict consensus of 4 trees: MPTs
/---------------------------------------------------------------------------------------------------------------------------------- Protorothyris
|
|       /-------------------------------------------------------------------------------------------------------------------------- Paleothyris
|       |
|       |       /------------------------------------------------------------------------------------------------------------------ Thuringothyris
|       |       |
|       |       |       /---------------------------------------------------------------------------------------------------------- Euconcordia
|       |       |       |
\-------+       |       |                                                                                                 /-------- Romeria prima
        |       |       |                                                                                         /-------+
        |       |       |                                                                                         |       \-------- Romeria texana
        |       |       |                                                                                 /-------+
        |       |       |                                /------------------------------------------------+       \---------------- Reiszorhinus
        |       |       |                                |                                                |
        \-------+       |                                |                                                \------------------------ Protocaptorhinus
                |       |                                |
                |       |                                |                                                                /-------- Ca. laticeps
                |       |                                |                                                                |
                |       |                        /-------+       /--------------------------------------------------------+-------- Ca. aguti
                |       |                        |       |       |                                                        |
                |       |                        |       |       |                                                        \-------- Ca. magnus
                |       |                        |       |       |
                |       |                        |       |       |       /--------------------------------------------------------- Labidosaurus
                |       |                        |       |       |       |
                \-------+                        |       \-------+       |                                                /-------- Labidosaurikos
                        |                        |               |       |                                        /-------+
                        |                        |               |       |                                        |       \-------- Rothianiscus
                        |                        |               |       |                                        |
                        |                        |               |       |                                        |       /-------- Captorhinikos
                        |                        |               |       |                                /-------+-------+
                        |                /-------+               \-------+                                |       |       \-------- Sumidadectes
                        |                |       |                       |                        /-------+       |
                        |                |       |                       |                        |       |       \---------------- Gansurhinus
                        |                |       |                       |                        |       |
                        |                |       |                       |               /--------+       \------------------------ Moradisaurus
                        |                |       |                       |               |        |
                        |                |       |                       |       /-------+        \-------------------------------- Indosauriscus
                        |        /-------+       |                       |       |       |
                        |        |       |       |                       \-------+       \----------------------------------------- Tramuntanasaurus
                        |        |       |       |                               |
                        |        |       |       |                               \------------------------------------------------- Rhodotheratus
                        |        |       |       |
                        \--------+       |       \--------------------------------------------------------------------------------- Saurorictus
                                 |       |
                                 |       \----------------------------------------------------------------------------------------- Rhiodenticulatus
                                 |
                                 \------------------------------------------------------------------------------------------------ Opisthodontosaurus

paup> hsearch keep=237;

Heuristic search settings:
  Optimality criterion = parsimony
    Character-status summary:
      Of 79 total characters:
        7 characters are of type 'ord' (Wagner)
        72 characters are of type 'unord'
        All characters have equal weight
        All characters are parsimony-informative
    Gaps are treated as "missing"
    Multistate taxa interpreted as uncertainty
  Starting tree(s) obtained via stepwise addition
    Addition sequence: simple (reference taxon = Protorothyris)
    Number of trees held at each step = 1
  Branch-swapping algorithm: tree-bisection-reconnection (TBR) with reconnection limit =
8
    Steepest descent option not in effect
  Initial 'Maxtrees' setting = 100
```

```
  Branches collapsed (creating polytomies) if maximum branch length is zero
  Keeping all trees less than or equal to score 237
  'MulTrees' option in effect
  No topological constraints in effect
  Trees are unrooted

Maxtrees reset to 100000

Heuristic search completed
  Total number of rearrangements tried = 256706293
  Score of best tree(s) found = 234
  Number of trees retained = 73487
  Time used = 00:01:28 (CPU time = 00:01:20.9)

paup> contree;

Strict consensus of 73487 trees: MPTs + 1 step
```

```
/------------------------------------------------------------------------------------------------------------------------------- Protorothyris
|
|                               /----------------------------------------------------------------------------------------------- Paleothyris
|                               |
|                               |                               /--------------------------------------------------------------- Thuringothyris
|                               |                               |
|                               |                               |                               /------------------------------- Euconcordia
|                               |                               |                               |
|                               |                               |                               +------------------------------- Romeria prima
\-------------------------------+                               |                               |
                                |                               |                               +------------------------------- Romeria texana
                                |                               |                               |
                                |                               |                               +------------------------------- Protocaptorhinus
                                |                               |                               |
                                |                               |                               +------------------------------- Rhiodenticulatus
                                \-------------------------------+                               |
                                                                |                               +------------------------------- Sauroricus
                                                                |                               |
                                                                |                               +------------------------------- Ca. laticeps
                                                                |                               |
                                                                |                               +------------------------------- Ca. aguti
                                                                |                               |
                                                                |                               +------------------------------- Ca. magnus
                                                                |                               |
                                                                |                               +------------------------------- Labidosaurus
                                                                |                               |
                                                                \-------------------------------+------------------------------- Labidosaurikos
                                                                                                |
                                                                                                +------------------------------- Moradisaurus
                                                                                                |
                                                                                                +------------------------------- Rothianiscus
                                                                                                |
                                                                                                +------------------------------- Captorhinikos
                                                                                                |
                                                                                                +------------------------------- Gansurhinus
                                                                                                |
                                                                                                +------------------------------- Sumidadectes
                                                                                                |
                                                                                                +------------------------------- Reiszorhinus
                                                                                                |
                                                                                                +------------------------------ Opisthodontosaurus
                                                                                                |
                                                                                                +------------------------------- Rhodotheratus
                                                                                                |
                                                                                                +------------------------------- Tramuntanasaurus
                                                                                                |
                                                                                                \------------------------------- Indosauriscus
```

```
paup> hsearch keep=238;

Heuristic search settings:
  Optimality criterion = parsimony
    Character-status summary:
      Of 79 total characters:
        7 characters are of type 'ord' (Wagner)
        72 characters are of type 'unord'
        All characters have equal weight
        All characters are parsimony-informative
    Gaps are treated as "missing"
    Multistate taxa interpreted as uncertainty
  Starting tree(s) obtained via stepwise addition
    Addition sequence: simple (reference taxon = Protorothyris)
    Number of trees held at each step = 1
  Branch-swapping algorithm: tree-bisection-reconnection (TBR) with reconnection limit =
8
    Steepest descent option not in effect
  Initial 'Maxtrees' setting = 100000
  Branches collapsed (creating polytomies) if maximum branch length is zero
  Keeping all trees less than or equal to score 238
  'MulTrees' option in effect
  No topological constraints in effect
  Trees are unrooted

Maxtrees reset to 10000000
```

Supplemental 2

```
Heuristic search completed
  Total number of rearrangements tried = 1220167335
  Score of best tree(s) found = 234
  Number of trees retained = 351746
  Time used = 00:09:30 (CPU time = 00:08:03.7)

paup> contree;
```

Strict consensus of 351746 trees: MPTs + 2 steps

```
/------------------------------------------------------------------------------------------------------------------------------------ Protorothyris
|
|                                                                 /------------------------------------------------------------------ Paleothyris
|                                                                 |
|                                                                 +------------------------------------------------------------------ Thuringothyris
|                                                                 |
|                                                                 +------------------------------------------------------------------ Euconcordia
|                                                                 |
|                                                                 +------------------------------------------------------------------ Romeria prima
|                                                                 |
|                                                                 +------------------------------------------------------------------ Romeria texana
|                                                                 |
|                                                                 +------------------------------------------------------------------ Protocaptorhinus
|                                                                 |
|                                                                 +------------------------------------------------------------------ Rhiodenticulatus
|                                                                 |
|                                                                 +------------------------------------------------------------------ Saurorictus
|                                                                 |
|                                                                 +------------------------------------------------------------------ Ca. laticeps
|                                                                 |
|                                                                 +------------------------------------------------------------------ Ca. aguti
|                                                                 |
|                                                                 +------------------------------------------------------------------ Ca. magnus
|                                                                 |
\-----------------------------------------------------------------+------------------------------------------------------------------ Labidosaurus
                                                                  |
                                                                  +------------------------------------------------------------------ Labidosaurikos
                                                                  |
                                                                  +------------------------------------------------------------------ Moradisaurus
                                                                  |
                                                                  +------------------------------------------------------------------ Rothianiscus
                                                                  |
                                                                  +------------------------------------------------------------------ Captorhinikos
                                                                  |
                                                                  +------------------------------------------------------------------ Gansurhinus
                                                                  |
                                                                  +------------------------------------------------------------------ Sumidadectes
                                                                  |
                                                                  +------------------------------------------------------------------ Reiszorhinus
                                                                  |
                                                                  +----------------------------------------------------------------- Opisthodontosaurus
                                                                  |
                                                                  +------------------------------------------------------------------ Rhodotheratus
                                                                  |
                                                                  +------------------------------------------------------------------ Tramuntanasaurus
                                                                  |
                                                                  \------------------------------------------------------------------ Indosauriscus
```

Phylogenetic characters

(1) Premaxilla: ventral margin aligned anteroposteriorly in lateral view (0); ventral margin aligned anteroventrally in lateral view (1).
(2) Premaxilla: alary process absent (0); alary process present on posterodorsal process (1).
(3) Premaxillary dentition: first tooth relatively small relative to maxillary caniniform (0); subequal to maxillary caniniform (1). In taxa lacking maxillary caniniforms, state 1 applies when the first premaxillary tooth is the largest marginal tooth present.
(4) Maxilla: relatively straight (0); posterior end flexed laterally (1).
(5) Maxilla: posterior-most tooth positioned at level of posterior margin of orbit (0) or positioned more anteriorly (1).
(6) Maxillary dentition: tooth stations number 30 teeth or more (0); teeth number 18–26 (1); teeth number 14–17 (2); teeth number 13 or less (3). For multiple-rowed taxa, only those teeth with unobstructed profiles when viewed laterally are considered. ORDERED
(7) Teeth on maxillary dental field: none (0); less than 40 (1); 40 or more (2). Definition modified from Reisz et al. (2011) for clarity. ORDERED
(8) Maxillary caniniform teeth: present (0); absent (1).
(9) Number of tooth rows in the upper jaw: one (0); two to four (1); five (2); six or more (3). ORDERED
(10) Marginal dentition: ‘cheek’ teeth recurved (0); chisel-shaped (1); bulbous and ogival (2); bulbous at base and conical above (3). ORDERED
(11) Maxilla: double row of teeth extend far anteriorly on tooth row absent (0); double row of teeth extend far anteriorly on tooth row present (1).
(12) Dental tooth wear: absent (0); present, modest (1); present, saddle shaped (2). ORDERED
(13) Lacrimal: suture with jugal small (0); well developed (1).
(14) Nasolacrimal suture: straight (0); interdigitating (1).

(15) Snout: broad, equal to or greater than 35% of skull length (0); narrow, equal to or less than 25% of skull length (1).
(16) Antorbital/cheek region: mainly formed by lacrimal and prefrontal (0); mostly formed by lacrimal due to dorsal expansion of the bone (1).
(17) Prefrontal: prefrontal-nasal suture shorter than lacrimal-nasal suture (0); prefrontal-nasal suture longer than lacrimal-nasal suture (1).
(18) Frontal: anterior process short, less than 40% of the frontal sagittal length (0); anterior process long, approximately 55% of the frontal sagittal length (1).
(19) Jugal: subtemporal process dorsoventrally low, equal to or less than 25% of skull height through orbital midpoint (0); subtemporal process dorsoventrally deep, at least 40% of skull height through orbit (1).
(20) Jugal: anterior extent ends posterior to anterior orbital margin (0); anterior extent reaches beyond anterior orbital margin (1).
(21) Jugal: postorbital extent shorter than remaining anterior extent (0); postorbital extent equal or longer (1).
(22) Quadratojugal: anteroposteriorly elongate (0); short, not extending anteriorly beyond midpoint of postorbital margin (1).
(23) Quadratojugal: acuminate anteriorly (0); square-tipped anteriorly (1).
(24) Quadratojugal: expanded dorsally (0); posteriorly straight or decreasing in height (1).
(25) Quadratojugal: maximum height approximately equal to one-third or less that of squamosal (0); nearly equal to half of squamosal height (1).
(26) Postorbital cheek: relatively straight (0); moderately expanded laterally (1); greatly expanded laterally (2).
(27) Parietal: distinct anterolateral process present that partially separates postfrontal and postorbital (0); does not strongly project between postfrontal and postorbital (1).
(28) Pineal foramen: positioned at midpoint of interparietal suture (0); anterior to midpoint of interparietal suture (1); posterior to midpoint of interparietal suture (2).
(29) Sculpturing: skull surface relatively smooth, with only small honeycombing pits or grooves (0); with pits and grooves with notably larger, randomly positioned pits on posterior skull table (1); low ridge-and-pit system with tiny pits and furrows (2).
(30) Supratemporal: obliquely oriented into anteromedial direction, thereby lying within a facet of the parietal (0); positioned mediolaterally at the posterior edge of the parietal (1).
(31) Supratemporal: small, slender element (0); large, contributing to skull table (1).
(32) Supratemporal-postparietal contact: tenuous or absent (0); well developed (1).
(33) Postparietal: contacts mate dorsally only, postparietals separated slightly ventrally by supraoccipital (0); contacts mate fully along height (1).
(34) Postparietal: transversely short with tabular present (0); transversely elongate with tabular absent (1).
(35) Postparietal: contribution to skull table absent or forms only narrow edge (0); contribution to skull table large, forming sculptured posterior portion of skull table (1).
(36) Skull table occipital margin: embayed bilaterally (0); straight (1); with single median embayment (2).
(37) Vomer: denticulated (0); edentulous (1).
(38) Vomer-pterygoid contact: extensive, at least 50% median border of vomer (0); short, no more than 33% median border of vomer (1).
(39) Palatine: denticulated (0); edentulous (1).
(40) Jugal/ectopterygoid: ectopterygoid present and alary process absent (0); ectopterygoid absent and alary process present, but no higher than the midpoint of the suborbital process of the jugal and distinct from the orbital margin (1); ectopterygoid absent and alary process present and positioned dorsally on the medial surface of the jugal, flush with orbital margin (2).
(41) Dentition on palatal ramus of pterygoid: present (0); greatly reduced or absent (1).
(42) Pterygoid: transverse flange dentition consists of shagreen of denticles (0); transverse flange dentition consists of at least one row of functional teeth (1); transverse flange dentition absent (2).
(43) Pterygoid: transverse flange broad-based and distinctly angular in ventral view (0); transverse flange narrow and tongue-like in ventral view (1).
(44) Suborbital foramen: absent (0); present (1); anteroposteriorly elongate (2).
(45) Parasphenoid: cultriform process extends anteriorly (0); cultriform process extends slightly dorsally at roughly 15º to the basal plane (1); cultriform process extends anterodorsally at more than 45º to the basal plane (2). ORDERED
(46) Parasphenoid: edentulous (0); denticulated (1).
(47) Parasphenoid: deep ventral groove between cristae ventrolateralis absent (0); deep ventral groove between cristae ventrolateralis present (1).
(48) Parasphenoid: posterolateral wing narrow, meets narrow head of stapes (0); wing broad, meets large head of stapes, in an elongate, nearly parasagittal suture (1).

(49) Opisthotic: paroccipital process long, extending near to medial edge of squamosal (0); process short, extending only slightly beyond body of opisthotic (1).
(50) Paroccipital process: broad (0); narrow (1).
(51) Stapes: distal process short (0); distal process elongate (1).
(52) Supraoccipital: slopes anterodorsally (0); aligned vertically or slopes posterodorsally (1).
(53) Supraoccipital: lateral ascending processes account for half or less height of bone (0); lateral ascending processes account two-thirds or more height of the bone (1).
(54) Occipital condyle: at level of quadrate condyles (0); immediately anterior to condyles (1).
(55) Exoccipital: lateral process on dorsal ramus absent (0); lateral process present (1).
(56) Dentary: Anterior dentary teeth: teeth uniformly small (0), multiple enlarged teeth present anteriorly (1); single enlarged tooth present anteriorly (2).
(57) Dentary: first tooth oriented mainly vertically (0); first tooth leans strongly anteriorly (1).
(58) Dentary: lingual shelf absent (0) or present (1).
(59) Mandibular ramus: relatively straight in ventral view (0); sigmoidal in ventral view (1).
(60) Mandibular ramus: narrow, 8% or less of total jaw length (0); broad, no less than 14% of total jaw length (1).
(61) Mandibular ramus: posterior end rectilinear in lateral view (0) or acuminate in lateral view (1).
(62) Mandibular ramus: lateral shelf absent (0); lateral shelf present below coronoid process (1).
(63) Coronoid: anterior process short (0); elongate (1).
(64) Meckelian foramen: small, anteroposterior length roughly 9% or less of total jaw length (0); large, anteroposterior length greater than or equal to 14% of total jaw length (1).
(65) Coronoid: posterodorsal process slender, forms dorsal-most quarter of lateral wall of adductor fossa (0); posterodorsal process deep, forms dorsal-most third of lateral wall of adductor fossa (1).
(66) Retroarticular process: absent (0); present and broader transversely than long (1); present and longer anteroposteriorly than broad (2).
(67) Dorsal vertebrae interzygophyseal width/centrum length: less than 1 (0), greater than 1, equal to or less than 2 (1), greater than 2 (2). Modified. ORDERED
(68) Neural spines of dorsal vertebrae: height or shape alternation absent (0); height or shape alternation present (1).
(69) Anterior dorsal centra: strongly ventrolaterally constricted (0); not strongly ventrolaterally constricted (1).
(70) Sacral ribs: first and second sacral rib roughly equal in size (0); first sacral rib larger than second rib (1).
(71) Ilium: iliac blade expanded dorsally (0); iliac blade narrow dorsally (1).
(72) Stylo- and zeugopodium: shaft slender and heads only moderately expanded (0); shaft massive and with proximal and distal heads significantly expanded, resulting in an overall stout impression (1).
(73) Humerus: supinator process parallel to shaft (0); supinator process absent (1). Modified slightly from Modesto et al. (2014).
(74) Manus and pes: elements long and slender (0); elements short and broad (1).
(75) Fourth metatarsal: less than half the length of tibia (0); more than half the length of tibia (1).
(76) First metacarpal: more than half the length of fourth metacarpal (0); less than half the length of fourth metacarpal (1).
(77) Skull length: <5 cm anteroposteriorly in mature specimens (0); >5 cm anteroposteriorly in mature specimens (1). From Albright et al. (2021).
(78) Distal end of tibia vesus proximal end or distal end of fibula: smaller (0); equal or larger (1). New.
(79) Alignment of multiple-tooth-row with respect to long axis of tooth battery: oblique, irregular (1); parallel to subparallel, straight (2). New. Taxa with a single row of teeth in maxilla/dentary are coded as ‘?’
